# Differential Colorectal Cancer Mortality Across Racial and Ethnic Groups: Impact of Socioeconomic Status, Clinicopathology, and Treatment‐Related Factors

**DOI:** 10.1002/cam4.70612

**Published:** 2025-03-04

**Authors:** Pierre Fwelo, Toluwani E. Adekunle, Tiwaladeoluwa B. Adekunle, Ella R. Garza, Emily Huang, Wayne R. Lawrence, Aldenise P. Ewing

**Affiliations:** ^1^ Department of Epidemiology, Human Genetics and Environmental Sciences UTHealth School of Public Health Houston Texas USA; ^2^ Department of Psychology, Public Health Program Calvin University School of Health Grand Rapids Michigan USA; ^3^ Center for Education in Health Sciences, Institute for Public Health and Medicine Northwestern University Feinberg School of Medicine Chicago Illinois USA; ^4^ Department of Surgery, College of Medicine The Ohio State University Columbus Ohio USA; ^5^ Division of Cancer Epidemiology and Genetics, National Cancer Institute National Institutes of Health Rockville Maryland USA; ^6^ Division of Epidemiology, College of Public Health The Ohio State University Columbus Ohio USA

**Keywords:** clinicopathologic factors, colorectal cancer, mediation analysis, mortality, racial and ethnic disparities, socioeconomic status, treatment variations

## Abstract

**Introduction:**

Non‐Hispanic Black (Black) colorectal cancer (CRC) patients have a higher risk of mortality than most other racial/ethnic groups. Limited studies examine the contribution of socioeconomic (SES), clinicopathologic, or treatment variations to mortality disparities. This retrospective cohort investigation examined the extent to which SES, clinicopathologic, and treatment factors explain racial/ethnic differences in CRC mortality.

**Methods:**

We studied 146,515 individuals, 18+ years old, with a confirmed diagnosis of CRC within 2010–2017, identified from the Surveillance, Epidemiology, and End Results (SEER) database. We performed Cox regression analyses to examine the association of race and ethnicity, surgery type, and tumor site with all‐cause mortality and CRC‐specific mortality. We then performed mediation analysis to quantify the extent to which mortality differences were mediated by SES, clinicopathologic, and treatment factors.

**Results:**

Black patients had a significantly higher hazard of all‐cause mortality than non‐Hispanic White (White) patients. The White versus Black patients' comparison demonstrated that variations in SES and clinicopathologic factors significantly explained 46.63% (indirect effect HR: 0.92, 95% CI 0.91–0.93) and 10.87% (indirect effect HR: 0.98, 95% CI 0.97–0.99) of the excess all‐cause mortality among Black patients, respectively. The Hispanic versus Black comparisons identified SES as the most influential mediator, explaining 19.68% of the excess all‐cause mortality. The proportions mediating for CRC‐specific mortality showed comparable outcomes to all‐cause mortality.

**Conclusion:**

Black patients had a greater risk for all‐cause mortality and CRC‐specific mortality attributed to SES and clinicopathologic variations compared to other racial/ethnic groups. Future studies should investigate equity in healthcare through interventions addressing SES‐related disparities.

## 1 Introduction

Racial and ethnic disparities in cancer survival due to tumor, sociodemographic, institutional, and neighborhood characteristics have been observed among US populations [1]. The burden of colorectal cancer (CRC)‐specific morbidity and mortality—the second leading cause of cancer‐related deaths in the United States—varies disproportionately based on patient characteristics, including age, sex, race, and ethnicity [2, 3, 4]. In the United States, non‐Hispanic, African American/Black (henceforth Black) adults have the highest CRC mortality rates compared to non‐Hispanic Caucasian/White (henceforth White) adults [5]. Despite extensive documentation of these race‐based disparities [4], few studies have explored the impact of other contributing factors, including socioeconomic status (SES), clinicopathologic characteristics, and treatment‐related factors, on CRC‐specific outcomes. Identifying the effects of these determinants may lead to intervention against the modifiable aspects of well‐known race‐based disparities in CRC‐specific morbidity and mortality.

Previous epidemiologic studies have associated patient‐level SES (i.e., education, income, or employment) with CRC‐related outcomes (e.g., patients who report earning a low income are more likely to experience worse surgical outcomes and greater risk of CRC‐related death compared with adults earning higher incomes) [6, 7]. Additionally, clinicopathologic characteristics (e.g., tumor grade, size, site, and stage) were previously reported to be associated with CRC disease severity and outcomes, including survival [8, 9]. Tumor location is associated with overall survival (e.g., patients diagnosed with rectal cancer report a higher 5‐year survival rate compared to patients diagnosed with colon cancer) [10]. Likewise, tumor‐sidedness was reported to be associated with survival. For instance, left‐sided tumors are associated with a lower risk of mortality [10, 11]. Similarly, larger tumors are associated with a lower likelihood of survival [12, 13].

Finally, treatment options are consequential for CRC‐related outcomes. Surgery, the most common treatment, cures up to half of patients [14]. Surgical intervention is contingent on tumor location and the presence or extent of metastasis [14, 15]. Patients may undergo surgery (radiofrequency ablation, cryosurgery, etc.) based on tumor size, stage, and histology [15]. However, when controlling for SES and stage at CRC diagnosis, current research suggests that Black, Hispanic, and Asian patients are less likely to receive surgery than their White counterparts [16]. Additionally, a prior study reported that racially minoritized men and older patients were more likely to refuse treatment, with stated reasons including associated costs of care or pre‐existing health conditions [17, 18].

Although racial and ethnic disparities in CRC‐specific outcomes have been extensively documented, understanding the contributing factors associated with racial and ethnic disparities is critical for advancing health equity. In this investigation, we quantified the extent to which SES, clinicopathologic, and treatment‐related factors may explain racial and ethnic differences in all‐cause and CRC‐specific mortality among individuals diagnosed with CRC.

## 2 Materials and Methods

### 2.1 Study Population and Data Source

We conducted a retrospective cohort investigation using secondary data derived from adults in the United States who received a primary diagnosis of CRC (i.e., encompassing the colon, sigmoid, or rectum) between 2010 and 2017. Data were obtained from SEER's specialized census tract‐level SES and rurality dataset [19]. Using SEER*stat 8.4.3, we identified 235,504 patients ≥ 18 years of age with a confirmed microscopic diagnosis of CRC as their primary diagnosis within the specified period. Of the 235,504 patients who met our inclusion criteria, our analysis sequentially excluded individuals with unknown surgical status (*n* = 22,345) and those who lacked essential information, including race and ethnicity, tumor grade, tumor stage, geographic location, tumor site, and tumor size (*n* = 66,644). Hence, our analysis included a sample size of 146, 515, which was adequately powered to answer the research questions (Table S2). The study population was de‐identified; thus, the study was exempted from approval by an Institutional Review Board.

#### 2.1.1 Outcomes

Our primary focus in this study encompassed all‐cause mortality and CRC‐specific mortality. Follow‐up survival time was defined as the time (in days) between the CRC date of diagnosis and the mortality event or last contact date (December 31, 2018), whichever occurred first. All‐cause mortality was defined as death from any cause. CRC‐specific mortality was defined as death due to CRC, and deaths due to other causes were censored.

#### 2.1.2 Exposures

Our study concentrated on three key exposure variables as predictors of mortality outcomes: race and ethnicity, tumor site, and surgery type. Race and ethnicity were categorized as non‐Hispanic Caucasian/White (henceforth White), non‐Hispanic African‐American/Black (henceforth Black), Hispanic (all racial groups), and non‐Hispanic (NH) Other (i.e., NH Asian or Pacific Islander and NH American Indian/Alaska Native) [20]. Tumor site was subdivided into cecum, ascending colon, transverse colon, descending colon, sigmoid colon, rectosigmoid junction, and rectum. Surgery type was classified into four categories: no surgery, local excision, segmental resection, and radical resection. Detailed information on surgery grouping is shown in Table S1. Additionally, SES, clinicopathologic characteristics (tumor stage, size, site, and grade), and treatment‐related variables (surgery type, chemotherapy, radiotherapy, and treatment delay) were the proposed factors mediating the association of race and ethnicity with mortality outcomes. Treatment delay was categorized as either “yes” or “no.” Patients who received their first course of treatment (surgery, chemotherapy, radiotherapy, or endocrine therapy) within 1 month of diagnosis were classified as “no,” while those who began treatment more than 3 months after diagnosis were classified as “yes” [21, 22, 23]. SES was comprised of Yost Index SES quintiles (lowest, lower middle, middle, higher middle, and highest SES) and rurality (all urban, mostly urban, mostly rural, and all rural). The Yost index is a comprehensive assessment of SES and is constructed by examining the primary factors derived from census tract‐level data, including adjusted median household income, median house value, median rent, the percentage of people living below 150% of the poverty line, an education index (i.e., percent with less than high school graduate, high school only and more than high school) and the percentages of working‐class and unemployed individuals within specific block groups [24, 25]. Rurality was defined according to the Census Bureau's percent of the population living in non‐urban areas and divided into four quartiles [26, 27]. We incorporated various cancer and sociodemographic characteristics as covariates based on empirical evidence [21, 23, 28]. These encompassed age, gender, marital status, and year of diagnosis.

### 2.2 Statistical Analysis

Descriptive statistics were calculated to describe the study population's sociodemographic and cancer‐related characteristics by surgery type, tumor site, race, and ethnicity. Significance testing was performed within each stratum using Pearson's chi‐squared test. We conducted a series of analyses to investigate the associations of surgery type, tumor site, and race and ethnicity with mortality outcomes (all‐cause mortality and CRC‐specific mortality). We generated crude hazard regression models and three sequentially adjusted Cox proportional hazard regression models. The first adjusted model incorporated SES, the second extended the adjustments to include tumor site and treatment characteristics, and the third model adjusted for the interaction of radiation therapy and chemotherapy with tumor site. We assessed the proportional hazard's assumption and allowed the violating variables (i.e., age and marital status) to have time‐varying effects by adjusting for the interaction between them and the natural log of survival time.

**FIGURE 1:**
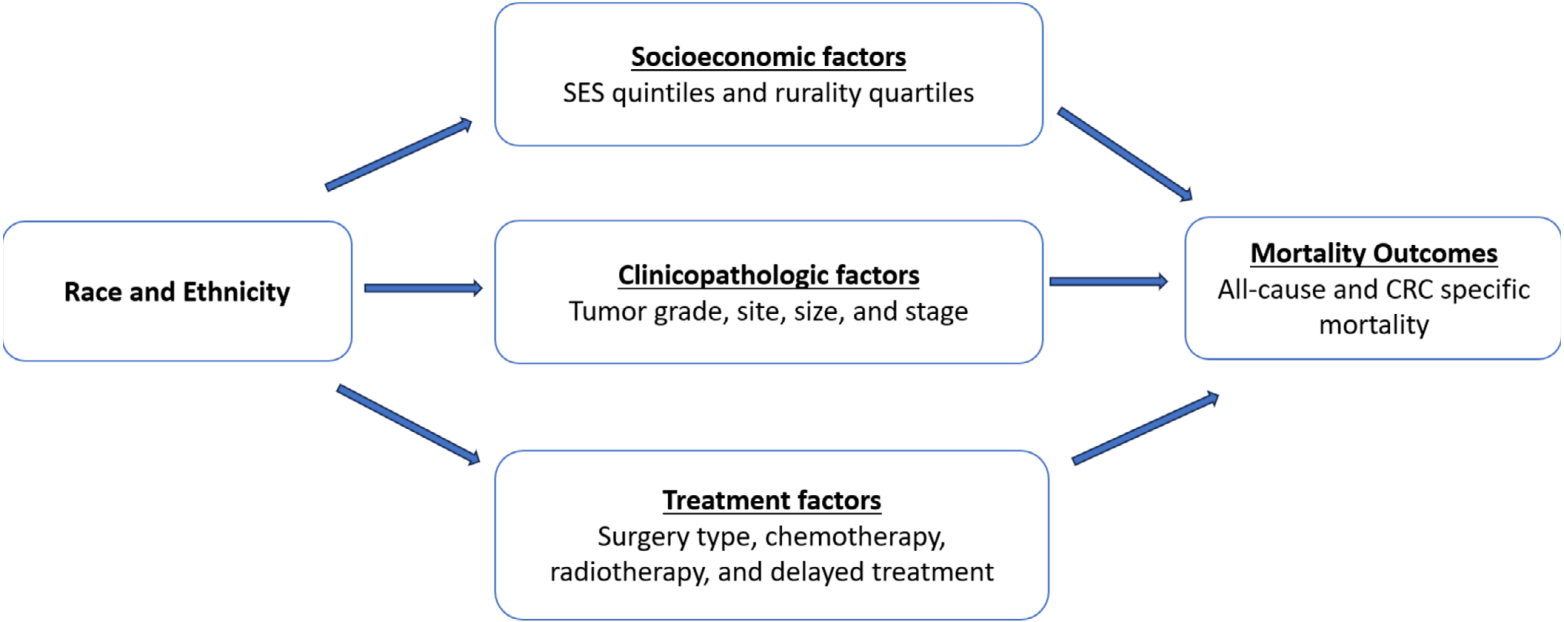
Proposed colorectal cancer mortality mediation model.

Moreover, we used the inverse odds weighting (IOW) method to perform mediation analyses and quantify the extent to which variations in SES, clinicopathologic characteristics, and treatment‐related factors explained the statistically significant differences in mortality outcomes between racial and ethnic groups [29, 30, 31, 32]. We employed a competing‐risk approach using Fine and Gray regression models, with non‐CRC‐specific mortality as competing events, to determine the risk of CRC‐specific mortality while employing conventional hazard models for all‐cause mortality [33, 34]. The mediation model we proposed to estimate the relative contribution of factors influenced by race and ethnicity and those that indirectly affected mortality outcomes is presented in Figure 1. We interpreted a statistically significant indirect effect as a corroboration of mediation. Lastly, we measured the extent of mediation by employing the proportion mediated, which represents the share of the mortality difference between racial and ethnic groups that can be attributed to the mediating factors [35, 36]. All statistical tests were two‐sided, and a *p*‐value less than 0.05 was considered statistically significant. Statistical analyses were conducted using SAS 9.4 and Stata 18.

## 3 Results

A total of 146,515 adults (mean [SD] age, 64.9 [14.1]) were included in the study. White patients constituted 66.49% of the total cohort, while Black, Hispanic, and NH Other patients comprised 11.79%, 12.23%, and 9.49%, respectively. When we examined the surgical procedures undergone by individuals, Black patients exhibited a higher percentage of “radical resection” (53.71%) compared to White (48.59%), Hispanic (44.67%), and NH Other (38.38%) patients (Table 1).

**TABLE 1:** Demographic and tumor characteristics in individuals diagnosed with colon cancer by race and ethnicity, 2010–2017.

	Total	White	Black	Hispanic	NH Other	*p* ^a^
*N* (%)	146,515 (100%)	97,413 (66.49%)	17,279 (11.79%)	17,915 (12.23%)	13,908 (9.49%)	
Surgery type						
No surgery	6069 (4.14%)	3940 (4.04%)	756 (4.38%)	840 (4.69%)	533 (3.83%)	< 0.001
Local excision	7136 (4.87%)	4155 (4.27%)	1110 (6.42%)	936 (5.22%)	935 (6.72%)	
Segmental resection	63,357 (43.24%)	41,985 (43.10%)	6133 (35.49%)	8137 (45.42%)	7102 (51.06%)	
Radical resection	69,953 (47.74%)	47,333 (48.59%)	9280 (53.71%)	8002 (44.67%)	5338 (38.38%)	
Tumor site						
Cecum	25,562 (17.45%)	17,748 (18.22%)	3432 (19.86%)	2847 (15.89%)	1535 (11.04%)	< 0.001
Ascending colon	22,549 (15.39%)	15,298 (15.70%)	3010 (17.42%)	2509 (14.01%)	1732 (12.45%)	
Transverse colon	10,342 (7.06%)	7099 (7.29%)	1305 (7.55%)	1087 (6.07%)	851 (6.12%)	
Descending colon	6641 (4.53%)	4065 (4.17%)	1032 (5.97%)	759 (4.24%)	785 (5.64%)	
Sigmoid colon	29,277 (19.98%)	18,495 (18.99%)	3146 (18.21%)	4064 (22.68%)	3572 (25.68%)	
Rectosigmoid junction	10,616 (7.25%)	6998 (7.18%)	961 (5.56%)	1448 (8.08%)	1209 (8.69%)	
Rectum	26,953 (18.40%)	17,709 (18.18%)	2608 (15.09%)	3540 (19.76%)	3096 (22.26%)	
Other	14,575 (9.95%)	10,001 (10.27%)	1785 (10.33%)	1661 (9.27%)	1128 (8.11%)	
Gender						
Female	71,361 (48.71%)	47,242 (48.50%)	8879 (51.39%)	8553 (47.74%)	6687 (48.08%)	< 0.001
Male	75,154 (51.29%)	50,171 (51.50%)	8400 (48.61%)	9362 (52.26%)	7221 (51.92%)	
Age at diagnosis						
< 45	10,687 (7.29%)	6026 (6.19%)	1345 (7.78%)	2230 (12.45%)	1086 (7.81%)	< 0.001
45–54	24,558 (16.76%)	14,566 (14.95%)	3633 (21.03%)	3759 (20.98%)	2600 (18.69%)	
55–64	35,025 (23.91%)	21,997 (22.58%)	5008 (28.98%)	4612 (25.74%)	3408 (24.50%)	
65–74	36,290 (24.77%)	24,762 (25.42%)	4177 (24.17%)	3896 (21.75%)	3455 (24.84%)	
75+	39,955 (27.27%)	30,062 (30.86%)	3116 (18.03%)	3418 (19.08%)	3359 (24.15%)	
SES						
Lowest	24,925 (17.01%)	12,093 (12.41%)	7462 (43.19%)	4204 (23.47%)	1166 (8.38%)	< 0.001
Lower‐mid	25,411 (17.34%)	16,265 (16.70%)	3495 (20.23%)	3954 (22.07%)	1697 (12.20%)	
Middle	27,267 (18.61%)	18,556 (19.05%)	2610 (15.11%)	3768 (21.03%)	2333 (16.77%)	
Upper‐mid	31,976 (21.82%)	22,913 (23.52%)	2233 (12.92%)	3313 (18.49%)	3517 (25.29%)	
Highest	36,936 (25.21%)	27,586 (28.32%)	1479 (8.56%)	2676 (14.94%)	5195 (37.35%)	
County of residence						
All rural	9545 (6.51%)	8547 (8.77%)	713 (4.13%)	170 (0.95%)	115 (0.83%)	< 0.001
Mostly rural	10,010 (6.83%)	8701 (8.93%)	774 (4.48%)	347 (1.94%)	188 (1.35%)	
Mostly urban	10,010 (6.83%)	8701 (8.93%)	774 (4.48%)	347 (1.94%)	188 (1.35%)	
All urban	29,186 (19.92%)	22,324 (22.92%)	3032 (17.55%)	2363 (13.19%)	1467 (10.55%)	
Marital status						
Married	82,396 (56.24%)	56,174 (57.67%)	6923 (40.07%)	10,189 (56.87%)	9110 (65.50%)	< 0.001
Single	26,239 (17.91%)	14,531 (14.92%)	5733 (33.18%)	3903 (21.79%)	2072 (14.90%)	
Divorced/Separated	16,307 (11.13%)	10,853 (11.14%)	2395 (13.86%)	1994 (11.13%)	1065 (7.66%)	
Widowed	21,573 (14.72%)	15,855 (16.28%)	2228 (12.89%)	1829 (10.21%)	1661 (11.94%)	
Tumor stage						
Localized	56,260 (38.40%)	38,071 (39.08%)	6513 (37.69%)	6490 (36.23%)	5186 (37.29%)	< 0.001
Regional by direct extension only	20,985 (14.32%)	14,481 (14.87%)	2121 (12.28%)	2598 (14.50%)	1785 (12.83%)	
Regional lymph nodes involved only	22,263 (15.20%)	14,259 (14.64%)	2876 (16.64%)	2835 (15.82%)	2293 (16.49%)	
Regional by both direct extension and lymph node involvement	23,275 (15.89%)	15,323 (15.73%)	2473 (14.31%)	2980 (16.63%)	2499 (17.97%)	
Distant	23,732 (16.20%)	15,279 (15.68%)	3296 (19.08%)	3012 (16.81%)	2145 (15.42%)	
Tumor grade						
Well differentiated; Grade I	16,199 (11.06%)	10,104 (10.37%)	2252 (13.03%)	2342 (13.07%)	1501 (10.79%)	< 0.001
Moderately differentiated; Grade II	102,571 (70.01%)	67,708 (69.51%)	12,343 (71.43%)	12,446 (69.47%)	10,074 (72.43%)	
Poorly differentiated; Grade III	22,740 (15.52%)	15,832 (16.25%)	2239 (12.96%)	2643 (14.75%)	2026 (14.57%)	
Undifferentiated; anaplastic; Grade IV	5005 (3.42%)	3769 (3.87%)	445 (2.58%)	484 (2.70%)	307 (2.21%)	
Tumor size						
0–1	12,668 (8.65%)	8076 (8.29%)	1716 (9.93%)	1485 (8.29%)	1391 (10%)	< 0.001
1.1–2	13,311 (9.09%)	8978 (9.22%)	1428 (8.26%)	1531 (8.55%)	1374 (9.88%)	
2.1–4	46,123 (31.48%)	31,234 (32.06%)	4995 (28.91%)	5281 (29.48%)	4613 (33.17%)	
4+	74,413 (50.79%)	49,125 (50.43%)	9140 (52.90%)	9618 (53.69%)	6530 (46.95%)	
Radiotherapy						
No	127,088 (86.74%)	84,351 (86.59%)	15,561 (90.06%)	15,288 (85.34%)	11,888 (85.48%)	< 0.001
Yes	19,427 (13.26%)	13,062 (13.41%)	1718 (9.94%)	2627 (14.66%)	2020 (14.52%)	
Chemotherapy						
No	84,483 (57.66%)	57,129 (58.65%)	9774 (56.57%)	9772 (54.55%)	7808 (56.14%)	< 0.001
Yes	62,032 (42.34%)	40,284 (41.35%)	7505 (43.43%)	8143 (45.45%)	6100 (43.86%)	
Year of diagnosis						
2010	18,095 (12.35%)	12,478 (12.81%)	2109 (12.21%)	1916 (10.69%)	1592 (11.45%)	< 0.001
2011	17,647 (12.04%)	12,036 (12.36%)	2055 (11.89%)	1966 (10.97%)	1590 (11.43%)	
2012	18,316 (12.50%)	12,398 (12.73%)	2130 (12.33%)	2118 (11.82%)	1670 (12.01%)	
2013	18,048 (12.32%)	12,117 (12.44%)	2144 (12.41%)	2092 (11.68%)	1695 (12.19%)	
2014	18,672 (12.74%)	12,370 (12.70%)	2228 (12.89%)	2282 (12.74%)	1792 (12.88%)	
2015	18,694 (12.76%)	12,278 (12.60%)	2188 (12.66%)	2430 (13.56%)	1798 (12.93%)	
2016	18,738 (12.79%)	12,085 (12.41%)	2232 (12.92%)	2534 (14.14%)	1887 (13.57%)	
2017	18,305 (12.49%)	11,651 (11.96%)	2193 (12.69%)	2577 (14.38%)	1884 (13.55%)	

^a^ Pearson chi‐squared test was performed to obtain the *p* values.

Radical resection constituted the most common surgical procedure at 47.74%. Local excision accounted for 4.87% of patients, with a majority being White adults (58.23%), aged 45–54 (25.83%). Patients who did not undergo surgery represented 4.14% of the total population, primarily consisting of White adults (64.92%), patients aged 55–64 (28.36%), and those in the highest SES (Table 2). Patients with distant metastasis (65.30%) represented most patients without surgery. The distribution of patients across tumor sites was as follows: cecum (17.45%), ascending colon (15.39%), transverse colon (7.06%), descending colon (4.53%), sigmoid colon (19.98%), rectosigmoid junction (7.25%), rectum (18.40%), other (9.95%). Among patients with tumors in the rectum, 58.73% were men and 41.27% were women.

**TABLE 2:** Patients and tumor characteristics in individuals diagnosed with colon cancer by surgical type and tumor site, 2010–2017.

	Surgical type				Tumor site							
	No surgery	Local excision	Segmental resection	Radical resection	Cecum	Ascending colon	Transverse colon	Descending colon	Sigmoid colon	Rectosigmoid junction	Rectum	Other
Race and ethnicity												
White	3940 (64.92%)	4155 (58.23%)	41,985 (66.27%)	47,333 (67.66%)	17,748 (69.43%)	15,298 (67.84%)	7099 (68.64%)	4065 (61.21%)	18,495 (63.17%)	6998 (65.92%)	17,709 (65.70%)	10,001 (68.62%)
Black	756 (12.46%)	1110 (15.55%)	6133 (9.68%)	9280 (13.27%)	3432 (13.43%)	3010 (13.35%)	1305 (12.62%)	1032 (15.54%)	3146 (10.75%)	961 (9.05%)	2608 (9.68%)	1785 (12.25%)
Hispanic	840 (13.84%)	936 (13.12%)	8137 (12.84%)	8002 (11.44%)	2847 (11.14%)	2509 (11.13%)	1087 (10.51%)	759 (11.43%)	4064 (13.88%)	1448 (13.64%)	3540 (13.13%)	1661 (11.40%)
NH Other	533 (8.78%)	935 (13.10%)	7102 (11.21%)	5338 (7.63%)	1535 (6.01%)	1732 (7.68%)	851 (8.23%)	785 (11.82%)	3572 (12.20%)	1209 (11.39%)	3096 (11.49%)	1128 (7.74%)
Surgery type												
No surgery	—	—	—	—	408 (1.60%)	339 (1.50%)	170 (1.64%)	97 (1.46%)	736 (2.51%)	493 (4.64%)	3601 (13.36%)	225 (1.54%)
Local excision	—	—	—	—	70 (0.27%)	129 (0.57%)	104 (1.01%)	164 (2.47%)	1188 (4.06%)	259 (2.44%)	4877 (18.09%)	345 (2.37%)
Segmental resection	—	—	—	—	4115 (16.10%)	3234 (14.34%)	3962 (38.31%)	2165 (32.60%)	21,959 (75%)	9234 (86.98%)	13,937 (51.71%)	4751 (32.60%)
Radical resection	—	—	—	—	20,969 (82.03%)	18,847 (83.58%)	6106 (59.04%)	4215 (63.47%)	5394 (18.42%)	630 (5.93%)	4538 (16.84%)	9254 (63.49%)
Tumor site												
Cecum	408 (6.72%)	70 (0.98%)	4115 (6.49%)	20,969 (29.98%)	—	—	—	—	—	—	—	—
Ascending colon	339 (5.59%)	129 (1.81%)	3234 (5.10%)	18,847 (26.94%)	—	—	—	—	—	—	—	—
Transverse colon	170 (2.80%)	104 (1.46%)	3962 (6.25%)	6106 (8.73%)	—	—	—	—	—	—	—	—
Descending colon	97 (1.60%)	164 (2.30%)	2165 (3.42%)	4215 (6.03%)	—	—	—	—	—	—	—	—
Sigmoid colon	736 (12.13%)	1188 (16.65%)	21,959 (34.66%)	5394 (7.71%)	—	—	—	—	—	—	—	—
Rectosigmoid junction	493 (8.12%)	259 (3.63%)	9234 (14.57%)	630 (0.90%)	—	—	—	—	—	—	—	—
Rectum	3601 (59.33%)	4877 (68.34%)	13,937 (22%)	4538 (6.49%)	—	—	—	—	—	—	—	—
Other	225 (3.71%)	345 (4.83%)	4751 (7.50%)	9254 (13.23%)	—	—	—	—	—	—	—	—
Gender												
Female	2459 (40.52%)	3308 (46.36%)	29,485 (46.54%)	36,109 (51.62%)	14,244 (55.72%)	12,176 (54%)	5302 (51.27%)	3091 (46.54%)	13,360 (45.63%)	4710 (44.37%)	11,123 (41.27%)	7355 (50.46%)
Male	3610 (59.48%)	3828 (53.64%)	33,872 (53.46%)	33,844 (48.38%)	11,318 (44.28%)	10,373 (46%)	5040 (48.73%)	3550 (53.46%)	15,917 (54.37%)	5906 (55.63%)	15,830 (58.73%)	7220 (49.54%)
Age at diagnosis												
< 45	523 (8.62%)	655 (9.18%)	5351 (8.45%)	4158 (5.94%)	976 (3.82%)	867 (3.84%)	572 (5.53%)	566 (8.52%)	2320 (7.92%)	917 (8.64%)	2487 (9.23%)	1982 (13.60%)
45–54	1229 (20.25%)	1843 (25.83%)	12,239 (19.32%)	9247 (13.22%)	2761 (10.80%)	2324 (10.31%)	1221 (11.81%)	1236 (18.61%)	6132 (20.94%)	2227 (20.98%)	6485 (24.06%)	2172 (14.90%)
55–64	1721 (28.36%)	1971 (27.62%)	16,212 (25.59%)	15,121 (21.62%)	5234 (20.48%)	4441 (19.69%)	2094 (20.25%)	1615 (24.32%)	7668 (26.19%)	3003 (28.29%)	7767 (28.82%)	3203 (21.98%)
65–74	1359 (22.39%)	1484 (20.80%)	15,124 (23.87%)	18,323 (26.19%)	6813 (26.65%)	6274 (27.82%)	2738 (26.47%)	1642 (24.73%)	7009 (23.94%)	2496 (23.51%)	5928 (21.99%)	3390 (23.26%)
75+	1237 (20.38%)	1183 (16.58%)	14,431 (22.78%)	23,104 (33.03%)	9778 (38.25%)	8643 (38.33%)	3717 (35.94%)	1582 (23.82%)	6148 (21%)	1973 (18.59%)	4286 (15.90%)	3828 (26.26%)
SES												
Lowest	1087 (17.91%)	1181 (16.55%)	10,051 (15.86%)	12,606 (18.02%)	4286 (16.77%)	3933 (17.44%)	1778 (17.19%)	1272 (19.15%)	5104 (17.43%)	1761 (16.59%)	4337 (16.09%)	2454 (16.84%)
Lower‐mid	1054 (17.37%)	1142 (16%)	10,846 (17.12%)	12,369 (17.68%)	4479 (17.52%)	3925 (17.41%)	1810 (17.50%)	1153 (17.36%)	5078 (17.34%)	1777 (16.74%)	4617 (17.13%)	2572 (17.65%)
Middle	1106 (18.22%)	1273 (17.84%)	11,897 (18.78%)	12,991 (18.57%)	4799 (18.77%)	4233 (18.77%)	1957 (18.92%)	1200 (18.07%)	5514 (18.83%)	1923 (18.11%)	4975 (18.46%)	2666 (18.29%)
Upper‐mid	1359 (22.39%)	1498 (20.99%)	14,032 (22.15%)	15,087 (21.57%)	5628 (22.02%)	4852 (21.52%)	2216 (21.43%)	1389 (20.92%)	6445 (22.01%)	2374 (22.36%)	5938 (22.03%)	3134 (21.50%)
Highest	1463 (24.11%)	2042 (28.62%)	16,531 (26.09%)	16,900 (24.16%)	6370 (24.92%)	5606 (24.86%)	2581 (24.96%)	1627 (24.50%)	7136 (24.37%)	2781 (26.20%)	7086 (26.29%)	3749 (25.72%)
Rurality												
All rural	373 (6.15%)	413 (5.79%)	3960 (6.25%)	4799 (6.86%)	1730 (6.77%)	1430 (6.34%)	724 (7%)	394 (5.93%)	1837 (6.27%)	651 (6.13%)	1827 (6.78%)	952 (6.53%)
Mostly rural	402 (6.62%)	411 (5.76%)	4406 (6.95%)	4791 (6.85%)	1742 (6.81%)	1517 (6.73%)	688 (6.65%)	413 (6.22%)	2014 (6.88%)	729 (6.87%)	1923 (7.13%)	984 (6.75%)
Mostly urban	1174 (19.34%)	1359 (19.04%)	12,390 (19.56%)	14,263 (20.39%)	5256 (20.56%)	4517 (20.03%)	2089 (20.20%)	1292 (19.45%)	5704 (19.48%)	2097 (19.75%)	5313 (19.71%)	2918 (20.02%)
All urban	4120 (67.89%)	4953 (69.41%)	42,601 (67.24%)	46,100 (65.90%)	16,834 (65.86%)	15,085 (66.90%)	6841 (66.15%)	4542 (68.39%)	19,722 (67.36%)	7139 (67.25%)	17,890 (66.37%)	9721 (66.70%)
Marital status												
Married	3211 (52.91%)	4302 (60.29%)	36,976 (58.36%)	37,907 (54.19%)	13,503 (52.82%)	12,253 (54.34%)	5747 (55.57%)	3681 (55.43%)	16,889 (57.69%)	6184 (58.25%)	16,144 (59.90%)	7995 (54.85%)
Single	1348 (22.21%)	1434 (20.10%)	11,429 (18.04%)	12,028 (17.19%)	4114 (16.09%)	3470 (15.39%)	1611 (15.58%)	1298 (19.55%)	5628 (19.22%)	1978 (18.63%)	5123 (19.01%)	3017 (20.70%)
Divorced/Separated	825 (13.59%)	715 (10.02%)	7051 (11.13%)	7716 (11.03%)	2855 (11.17%)	2364 (10.48%)	1075 (10.39%)	729 (10.98%)	3349 (11.44%)	1237 (11.65%)	3149 (11.68%)	1549 (10.63%)
Widowed	685 (11.29%)	685 (9.60%)	7901 (12.47%)	12,302 (17.59%)	5090 (19.91%)	4462 (19.79%)	1909 (18.46%)	933 (14.05%)	3411 (11.65%)	1217 (11.46%)	2537 (9.41%)	2014 (13.82%)
Tumor stage												
Localized	645 (10.63%)	6645 (93.12%)	24,141 (38.10%)	24,829 (35.49%)	9141 (35.76%)	9290 (41.20%)	4048 (39.14%)	2377 (35.79%)	10,809 (36.92%)	3557 (33.51%)	11,435 (42.43%)	5603 (38.44%)
Regional by direct extension only	470 (7.74%)	145 (2.03%)	9045 (14.28%)	11,325 (16.19%)	3611 (14.13%)	3526 (15.64%)	1841 (17.80%)	1077 (16.22%)	3705 (12.65%)	1446 (13.62%)	3260 (12.10%)	2519 (17.28%)
Regional lymph nodes involved only	450 (7.41%)	105 (1.47%)	11,152 (17.60%)	10,556 (15.09%)	3870 (15.14%)	3329 (14.76%)	1407 (13.60%)	1023 (15.40%)	4864 (16.61%)	1885 (17.76%)	4324 (16.04%)	1561 (10.71%)
Regional by both direct extension and lymph node involvement	541 (8.91%)	49 (0.69%)	10,434 (16.47%)	12,251 (17.51%)	4364 (17.07%)	3492 (15.49%)	1595 (15.42%)	1141 (17.18%)	4471 (15.27%)	1774 (16.71%)	4269 (15.84%)	2169 (14.88%)
Distant	3963 (65.30%)	192 (2.69%)	8585 (13.55%)	10,992 (15.71%)	4576 (17.90%)	2912 (12.91%)	1451 (14.03%)	1023 (15.40%)	5428 (18.54%)	1954 (18.41%)	3665 (13.60%)	2723 (18.68%)
Tumor grade												
Well differentiated; Grade I	443 (7.30%)	3234 (45.32%)	6534 (10.31%)	5988 (8.56%)	2197 (8.59%)	1781 (7.90%)	863 (8.34%)	589 (8.87%)	2779 (9.49%)	811 (7.64%)	4213 (15.63%)	2966 (20.35%)
Moderately differentiated; Grade II	4122 (67.92%)	3527 (49.43%)	47,262 (74.60%)	47,660 (68.13%)	17,036 (66.65%)	15,085 (66.90%)	7126 (68.90%)	4947 (74.49%)	22,584 (77.14%)	8176 (77.02%)	19,229 (71.34%)	8388 (57.55%)
Poorly differentiated; Grade III	1339 (22.06%)	320 (4.48%)	7934 (12.52%)	13,147 (18.79%)	5026 (19.66%)	4574 (20.28%)	1904 (18.41%)	910 (13.70%)	3268 (11.16%)	1368 (12.89%)	3046 (11.30%)	2644 (18.14%)
Undifferentiated; anaplastic; Grade IV	165 (2.72%)	55 (0.77%)	1627 (2.57%)	3158 (4.51%)	1303 (5.10%)	1109 (4.92%)	449 (4.34%)	195 (2.94%)	646 (2.21%)	261 (2.46%)	465 (1.73%)	577 (3.96%)
Tumor size												
0–1	122 (2.01%)	4436 (62.16%)	4877 (7.70%)	3233 (4.62%)	1144 (4.48%)	1278 (5.67%)	567 (5.48%)	460 (6.93%)	2464 (8.42%)	613 (5.77%)	4252 (15.78%)	1890 (12.97%)
1.1–2	270 (4.45%)	1317 (18.46%)	6540 (10.32%)	5184 (7.41%)	1638 (6.41%)	1706 (7.57%)	990 (9.57%)	559 (8.42%)	2823 (9.64%)	873 (8.22%)	3129 (11.61%)	1593 (10.93%)
2.1–4	1616 (26.63%)	921 (12.91%)	21,961 (34.66%)	21,625 (30.91%)	7502 (29.35%)	7110 (31.53%)	3444 (33.30%)	2256 (33.97%)	9996 (34.14%)	3507 (33.04%)	8234 (30.55%)	4074 (27.95%)
4+	4061 (66.91%)	462 (6.47%)	29,979 (47.32%)	39,911 (57.05%)	15,278 (59.77%)	12,455 (55.24%)	5341 (51.64%)	3366 (50.69%)	13,994 (47.80%)	5623 (52.97%)	11,338 (42.07%)	7018 (48.15%)
Radiotherapy												
No	3107 (51.19%)	6407 (89.78%)	52,184 (82.37%)	65,390 (93.48%)	25,200 (98.58%)	22,350 (99.12%)	10,257 (99.18%)	6542 (98.51%)	28,339 (96.80%)	8243 (77.65%)	11,747 (43.58%)	14,410 (98.87%)
Yes	2962 (48.81%)	729 (10.22%)	11,173 (17.63%)	4563 (6.52%)	362 (1.42%)	199 (0.88%)	85 (0.82%)	99 (1.49%)	938 (3.20%)	2373 (22.35%)	15,206 (56.42%)	165 (1.13%)
Chemotherapy												
No	492 (8.11%)	6356 (89.07%)	34,664 (54.71%)	42,971 (61.43%)	16,526 (64.65%)	15,544 (68.93%)	6881 (66.53%)	3993 (60.13%)	16,981 (58%)	5100 (48.04%)	9899 (36.73%)	9559 (65.58%)
Yes	5577 (91.89%)	780 (10.93%)	28,693 (45.29%)	26,982 (38.57%)	9036 (35.35%)	7005 (31.07%)	3461 (33.47%)	2648 (39.87%)	12,296 (42%)	5516 (51.96%)	17,054 (63.27%)	5016 (34.42%)
Year of diagnosis												
2010	510 (8.40%)	643 (9.01%)	7987 (12.61%)	8955 (12.80%)	3280 (12.83%)	2821 (12.51%)	1320 (12.76%)	828 (12.47%)	3680 (12.57%)	1434 (13.51%)	3119 (11.57%)	1613 (11.07%)
2011	601 (9.90%)	650 (9.11%)	7644 (12.06%)	8752 (12.51%)	3209 (12.55%)	2849 (12.63%)	1206 (11.66%)	826 (12.44%)	3590 (12.26%)	1326 (12.49%)	3026 (11.23%)	1615 (11.08%)
2012	661 (10.89%)	797 (11.17%)	7839 (12.37%)	9019 (12.89%)	3316 (12.97%)	2842 (12.60%)	1266 (12.24%)	832 (12.53%)	3664 (12.51%)	1385 (13.05%)	3302 (12.25%)	1709 (11.73%)
2013	721 (11.88%)	845 (11.84%)	7708 (12.17%)	8774 (12.54%)	3284 (12.85%)	2800 (12.42%)	1327 (12.83%)	817 (12.30%)	3568 (12.19%)	1275 (12.01%)	3306 (12.27%)	1671 (11.46%)
2014	850 (14.01%)	905 (12.68%)	7999 (12.63%)	8918 (12.75%)	3280 (12.83%)	2851 (12.64%)	1325 (12.81%)	850 (12.80%)	3746 (12.80%)	1356 (12.77%)	3511 (13.03%)	1753 (12.03%)
2015	919 (15.14%)	1063 (14.90%)	8119 (12.81%)	8593 (12.28%)	3120 (12.21%)	2851 (12.64%)	1318 (12.74%)	801 (12.06%)	3615 (12.35%)	1317 (12.41%)	3602 (13.36%)	2070 (14.20%)
2016	859 (14.15%)	1106 (15.50%)	8287 (13.08%)	8486 (12.13%)	3084 (12.06%)	2798 (12.41%)	1326 (12.82%)	835 (12.57%)	3744 (12.79%)	1314 (12.38%)	3565 (13.23%)	2072 (14.22%)
2017	948 (15.62%)	1127 (15.79%)	7774 (12.27%)	8456 (12.09%)	2989 (11.69%)	2737 (12.14%)	1254 (12.13%)	852 (12.83%)	3670 (12.54%)	1209 (11.39%)	3522 (13.07%)	2072 (14.22%)

### 3.1 All‐Cause Mortality

Black patients [adjusted hazard ratio (aHR): 1.08, 95% CI 1.05–1.11] had significantly higher risk of all‐cause mortality than White patients (model 3) (Table 3). Conversely, Hispanic patients (aHR: 0.94, 95% CI 0.91–0.97) and NH Other (aHR: 0.91, 95% CI 0.88–0.94) patients were at lower risk for all‐cause mortality compared to White patients (model 3). Patients who underwent a surgical procedure (i.e., local excision, segmental, and radical resection) were at lower risk for all‐cause mortality than patients with no surgery in the crude and adjusted models. Patients who underwent local excision (aHR: 0.43, 95% CI 0.35–0.54), segmental resection (aHR: 0.28, 95% CI 0.26–0.31), and radical resection (aHR: 0.29, 95% CI 0.27–0.33) were at lower risk for all‐cause mortality compared to people who did not undergo surgery (model 3). Among patients who did not receive radiotherapy or chemotherapy, those with the primary tumor located in the transverse colon (aHR: 1.17, 95% CI 1.06–1.30) were at higher risk for all‐cause mortality, whereas those with tumors in the rectum (aHR: 0.78, 95% CI 0.70–0.87) were at a significantly lower risk for all‐cause mortality compared with those with tumors located in the cecum. Additionally, patients in the lowest SES quintile compared to the highest, men compared to women, and single compared to married patients were all at higher risk for all‐cause mortality.

**TABLE 3:** Risk of all‐cause mortality among US adults diagnosed with colorectal cancer, 2010–2017.

	Overall mortality			
	Crude proportional hazard ratio (95% confidence interval)	Model 1^a^ proportional hazard ratio (95% confidence interval)	Model 2^b^ proportional hazard ratio (95% confidence interval)	Model 3^c^ proportional hazard ratio (95% confidence interval)
Race and ethnicity				
White	REF^d^	REF	REF	REF
Black	1.10 (1.07–1.13)	1.09 (1.05–1.12)	1.09 (1.05–1.12)	1.08 (1.05–1.11)
Hispanic	0.89 (0.87–0.92)	0.96 (0.93–0.99)	0.94 (0.91–0.97)	0.94 (0.91–0.97)
NH^e^ Other	0.82 (0.80–0.85)	0.95 (0.92–0.98)	0.91 (0.88–0.94)	0.91 (0.88–0.94)
Surgery type				
No surgery	REF	REF	REF	REF
Local excision	0.11 (0.11–0.12)	0.09 (0.08–0.11)	0.45 (0.36–0.56)	0.43 (0.35–0.54)
Segmental resection	0.22 (0.21–0.22)	0.15 (0.14–0.16)	0.28 (0.26–0.31)	0.28 (0.26–0.31)
Radical resection	0.28 (0.27–0.29)	0.17 (0.16–0.19)	0.29 (0.26–0.32)	0.29 (0.27–0.33)
Tumor site^f^				
Cecum	REF	REF	REF	REF
Ascending colon	0.87 (0.81–0.94)	0.88 (0.81–0.95)	1.03 (0.95–1.11)	1.05 (0.97–1.14)
Transverse colon	0.94 (0.85–1.04)	1.01 (0.91–1.11)	1.14 (1.03–1.26)	1.17 (1.06–1.30)
Descending colon	0.67 (0.59–0.76)	0.83 (0.73–0.94)	1.01 (0.88–1.15)	1.06 (0.93–1.21)
Sigmoid colon	0.51 (0.47–0.55)	0.67 (0.61–0.73)	0.75 (0.69–0.82)	0.80 (0.73–0.87)
Rectosigmoid junction	0.54 (0.49–0.61)	0.65 (0.58–0.74)	0.80 (0.70–0.90)	0.87 (0.77–0.99)
Rectum	0.42 (0.39–0.46)	0.34 (0.31–0.38)	0.69 (0.63–0.77)	0.78 (0.70–0.87)
Other	0.81 (0.74–0.89)	0.99 (0.91–1.09)	1.02 (0.93–1.12)	1.02 (0.93–1.12)
Gender				
Female	REF	REF	REF	REF
Male	0.92 (0.88–0.97)	1.15 (1.09–1.21)	1.17 (1.11–1.23)	1.17 (1.11–1.23)
Age at diagnosis^tvc^				
< 45	REF	REF	REF	REF
45–54	1.12 (0.97–1.30)	1.22 (1.05–1.41)	1.21 (1.04–1.41)	1.21 (1.04–1.40)
55–64	1.62 (1.41–1.86)	1.63 (1.42–1.88)	1.55 (1.35–1.78)	1.53 (1.33–1.77)
65–74	2.14 (1.87–2.45)	2.16 (1.88–2.48)	2.14 (1.86–2.46)	2.12 (1.84–2.44)
75+	4.36 (3.82–4.96)	4.08 (3.57–4.68)	3.23 (2.81–3.71)	3.20 (2.78–3.68)
SES				
Lowest	REF	REF	REF	REF
Lower‐mid	0.92 (0.89–0.94)	0.92 (0.89–0.95)	0.92 (0.89–0.94)	0.92 (0.89–0.94)
Middle	0.87 (0.84–0.89)	0.88 (0.85–0.91)	0.88 (0.86–0.91)	0.88 (0.86–0.91)
Upper‐mid	0.81 (0.79–0.84)	0.83 (0.80–0.85)	0.82 (0.80–0.85)	0.83 (0.80–0.85)
Highest	0.71 (0.69–0.73)	0.74 (0.72–0.76)	0.74 (0.72–0.76)	0.74 (0.72–0.76)
Rurality				
All rural	REF	REF	REF	REF
Mostly rural	0.95 (0.91–0.99)	1.03 (0.98–1.08)	1.04 (0.99–1.09)	1.04 (0.99–1.09)
Mostly urban	0.93 (0.90–0.97)	1.00 (0.96–1.04)	1.01 (0.97–1.05)	1.01 (0.97–1.06)
All urban	0.93 (0.89–0.96)	0.98 (0.95–1.02)	1.00 (0.96–1.04)	1.00 (0.97–1.04)
Marital status^tvc^				
Married	REF	REF	REF	REF
Single	1.43 (1.34–1.53)	1.61 (1.50–1.73)	1.36 (1.27–1.46)	1.36 (1.27–1.46)
Divorced/Separated	1.48 (1.36–1.60)	1.47 (1.36–1.60)	1.35 (1.24–1.46)	1.35 (1.24–1.46)
Widowed	2.49 (2.34–2.66)	1.57 (1.46–1.68)	1.31 (1.22–1.41)	1.31 (1.22–1.41)
Tumor stage				
Localized	REF		REF	REF
Regional by direct extension only	2.03 (1.83–2.24)		1.84 (1.66–2.05)	1.84 (1.66–2.05)
Regional lymph nodes involved only	2.06 (1.87–2.27)		3.67 (3.31–4.08)	3.68 (3.32–4.09)
Regional by both direct extension and lymph node involvement	3.66 (3.35–3.99)		5.52 (5.02–6.07)	5.52 (5.02–6.07)
Distant	10.60 (9.84–11.42)		16.90 (15.49–18.43)	16.76 (15.36–18.29)
Tumor grade				
Well differentiated; Grade I	REF		REF	REF
Moderately differentiated; Grade II	2.09 (1.86–2.35)		1.48 (1.31–1.67)	1.48 (1.31–1.67)
Poorly differentiated; Grade III	7.03 (6.22–7.93)		3.19 (2.82–3.61)	3.19 (2.82–3.62)
Undifferentiated; anaplastic; Grade IV	9.47 (8.20–10.93)		4.17 (3.61–4.83)	4.17 (3.61–4.83)
Tumor size				
0–1	REF		REF	REF
1.1–2	2.18 (1.77–2.68)		1.42 (1.15–1.76)	1.43 (1.15–1.77)
2.1–4	4.35 (3.64–5.19)		1.65 (1.36–1.99)	1.66 (1.37–2.00)
4+	8.47 (7.12–10.07)		2.13 (1.76–2.58)	2.15 (1.78–2.59)
Radiotherapy^g^				
No	REF		REF	REF
Yes	0.54 (0.50–0.59)		1.01 (0.98–1.05)	1.24 (1.09–1.42)
Chemotherapy^h^				
No	REF		REF	REF
Yes	0.58 (0.55–0.61)		0.18 (0.17–0.19)	0.20 (0.18–0.21)
Treatment delay				
No	REF		REF	REF
Yes	0.98 (0.96–1.01)		0.91 (0.89–0.94)	0.91 (0.89–0.94)
Tumor site for patients who underwent radiotherapy				
Radiotherapy and cecum				REF
Radiotherapy and ascending colon				1.14 (0.91–1.42)
Radiotherapy and transverse colon				1.19 (0.90–1.59)
Radiotherapy and descending colon				1.41 (1.05–1.91)
Radiotherapy and sigmoid colon				0.88 (0.75–1.04)
Radiotherapy and rectosigmoid colon				0.83 (0.71–0.97)
Radiotherapy and rectum				0.77 (0.67–0.89)
Radiotherapy and other				1.01 (0.79–1.29)
Tumor site for patients who underwent chemotherapy				
Chemotherapy and cecum				REF
Chemotherapy and ascending colon				0.92 (0.86–0.98)
Chemotherapy and transverse colon				0.90 (0.83–0.98)
Chemotherapy and descending colon				0.80 (0.73–0.88)
Chemotherapy and sigmoid colon				0.85 (0.80–0.91)
Chemotherapy and rectosigmoid colon				0.81 (0.75–0.88)
Chemotherapy and rectum				0.84 (0.78–0.90)
Chemotherapy and other				1.01 (0.94–1.08)

Abbreviation: Tvc = time‐varying covariate.

^a^ Model 1 = Associations of surgery type, tumor site, and race and ethnicity with mortality outcomes adjusted for sociodemographic factors.

^b^ Model 2 = Extended Model 1 by adjusting for tumor and treatment characteristics.

^c^ Model 3 = Extended Model 2 by adjusting for the interaction of radiation therapy and chemotherapy with tumor site.

^d^ Ref = Reference group.

^e^ NH = non‐Hispanic.

^f^ The values provided in Model 3 are when the patients did not undergo chemotherapy or radiotherapy.

^g^ The values provided in Model 3 represent radiotherapy for cecum cases only.

^h^ The values provided in Model 3 represent chemotherapy for cecum cases only.

### 3.2 Colorectal Cancer‐Specific Mortality

As shown in Table 4, Black patients (aHR: 1.14, 95% CI 1.10–1.18) were at significantly increased risk for CRC‐specific mortality than White patients (model 3). Moreover, patients who underwent local excision (aHR: 0.34, 95% CI 0.25–0.46), segmental resection (aHR: 0.24, 95% CI 0.21–0.26), or radical resection (aHR: 0.25, 95% CI 0.22–0.28) were less likely to experience CRC specific mortality than those who did not undergo surgery (Model 3). Among patients who only received radiotherapy (did not receive chemotherapy), patients with primary tumors situated in the descending colon (aHR: 1.46, 95% CI 1.06–2.01) demonstrated a higher likelihood of experiencing CRC‐specific mortality. Conversely, individuals with tumors in the rectum (aHR: 0.79, 95% CI 0.68–0.92) were notably at lower risk of CRC‐specific mortality than those with tumors in the cecum, as observed in Model 3. Patients who waited more than 1 month between diagnosis and the first course of treatment were less likely to have CRC‐specific mortality (aHR: 0.85, 95% CI 0.82–0.88) than those who received treatment within 1 month of diagnosis.

**TABLE 4:** Risk of colorectal cancer‐specific mortality among US adults diagnosed with colorectal cancer, 2010–2017.

	Colorectal cancer‐specific mortality			
	Crude proportional hazard ratio (95% confidence interval)	Model 1^a^ proportional hazard ratio (95% confidence interval)	Model 2^b^ proportional hazard ratio (95% confidence interval)	Model 3^c^ proportional hazard ratio (95% confidence interval)
Race and ethnicity				
White	REF^d^	REF	REF	REF
Black	1.21 (1.17–1.25)	1.15 (1.11–1.19)	1.15 (1.11–1.19)	1.14 (1.10–1.18)
Hispanic	1.04 (1.00–1.07)	1.06 (1.02–1.10)	1.04 (1.01–1.08)	1.04 (1.00–1.08)
NH^e^ other	0.93 (0.89–0.97)	1.05 (1.01–1.09)	1.00 (0.96–1.04)	1.00 (0.96–1.04)
Surgery type				
No surgery	REF	REF	REF	REF
Local excision	0.06 (0.05–0.06)	0.06 (0.04–0.08)	0.36 (0.26–0.49)	0.34 (0.25–0.46)
Segmental resection	0.17 (0.17–0.18)	0.11 (0.10–0.12)	0.24 (0.21–0.26)	0.24 (0.21–0.26)
Radical resection	0.22 (0.21–0.23)	0.13 (0.12–0.14)	0.24 (0.22–0.27)	0.25 (0.22–0.28)
Tumor site^f^				
Cecum	REF	REF	REF	REF
Ascending colon	0.86 (0.78–0.94)	0.86 (0.79–0.95)	1.05 (0.95–1.15)	1.05 (0.95–1.15)
Transverse colon	0.98 (0.87–1.10)	1.06 (0.94–1.19)	1.23 (1.09–1.38)	1.24 (1.10–1.40)
Descending colon	0.52 (0.45–0.61)	0.67 (0.57–0.79)	0.86 (0.73–1.01)	0.93 (0.79–1.10)
Sigmoid colon	0.43 (0.39–0.47)	0.57 (0.52–0.64)	0.67 (0.61–0.75)	0.74 (0.66–0.83)
Rectosigmoid junction	0.48 (0.42–0.55)	0.57 (0.49–0.66)	0.74 (0.64–0.86)	0.86 (0.74–0.99)
Rectum	0.35 (0.32–0.39)	0.26 (0.23–0.29)	0.59 (0.52–0.67)	0.74 (0.65–0.83)
Other	0.78 (0.70–0.87)	0.98 (0.88–1.10)	0.99 (0.89–1.10)	0.99 (0.89–1.11)
Gender				
Female	REF	REF	REF	REF
Male	0.85 (0.80–0.90)	1.12 (1.10–1.15)	1.14 (1.11–1.16)	1.11 (1.04–1.18)
Age at diagnosis^tvc^				
< 45	REF	REF	REF	REF
45–54	1.17 (0.99–1.38)	1.29 (1.08–1.52)	1.28 (1.08–1.52)	1.28 (1.08–1.52)
55–64	1.76 (1.50–2.06)	1.78 (1.52–2.09)	1.67 (1.42–1.96)	1.65 (1.40–1.94)
65–74	2.39 (2.05–2.79)	2.42 (2.07–2.83)	2.40 (2.05–2.82)	2.38 (2.03–2.79)
75+	5.58 (4.80–6.49)	5.16 (4.41–6.03)	3.87 (3.30–4.55)	3.83 (3.26–4.50)
SES				
Lowest	REF	REF	REF	REF
Lower‐mid	0.92 (0.89–0.96)	0.95 (0.92–0.99)	0.94 (0.91–0.98)	0.94 (0.91–0.98)
Middle	0.87 (0.84–0.90)	0.91 (0.88–0.94)	0.91 (0.88–0.94)	0.91 (0.88–0.94)
Upper‐mid	0.82 (0.79–0.84)	0.86 (0.83–0.89)	0.85 (0.82–0.89)	0.86 (0.83–0.89)
Highest	0.72 (0.70–0.75)	0.78 (0.76–0.81)	0.78 (0.75–0.81)	0.78 (0.75–0.81)
Rurality				
All rural	REF	REF	REF	REF
Mostly rural	0.96 (0.91–1.02)	1.00 (0.95–1.06)	1.02 (0.96–1.08)	1.02 (0.96–1.08)
Mostly urban	0.93 (0.89–0.98)	0.97 (0.93–1.02)	0.99 (0.94–1.04)	1.00 (0.95–1.04)
All urban	0.93 (0.89–0.97)	0.95 (0.91–0.99)	0.98 (0.94–1.03)	0.99 (0.94–1.03)
Marital status^tvc^				
Married	REF	REF	REF	REF
Single	1.42 (1.31–1.54)	1.62 (1.49–1.75)	1.34 (1.23–1.45)	1.32 (1.22–1.44)
Divorced/Separated	1.46 (1.33–1.61)	1.46 (1.33–1.61)	1.32 (1.20–1.45)	1.31 (1.19–1.44)
Widowed	2.83 (2.63–3.06)	1.63 (1.50–1.77)	1.33 (1.23–1.44)	1.31 (1.21–1.43)
Tumor stage				
Localized	REF		REF	REF
Regional by direct extension only	2.85 (2.47–3.30)		2.66 (2.29–3.10)	2.57 (2.21–3.00)
Regional lymph nodes involved only	2.64 (2.30–3.04)		5.14 (4.44–5.96)	5.05 (4.36–5.86)
Regional by both direct extension and lymph node involvement	5.55 (4.91–6.28)		9.10 (7.98–10.38)	8.74 (7.64–9.99)
Distant	20.79 (18.64–23.20)		36.92 (32.70–41.68)	34.69 (30.64–39.29)
Tumor grade				
Well differentiated; Grade I	REF		REF	REF
Moderately differentiated; Grade II	2.15 (1.85–2.51)		1.52 (1.30–1.77)	1.51 (1.29–1.76)
Poorly differentiated; Grade III	9.44 (8.07–11.03)		3.91 (3.34–4.59)	3.85 (3.28–4.52)
Undifferentiated; anaplastic; Grade IV	13.02 (10.91–15.55)		5.26 (4.40–6.30)	5.12 (4.28–6.14)
Tumor size				
0–1	REF		REF	REF
1.1–2	2.16 (1.59–2.92)		1.46 (1.32–1.61)	1.22 (0.90–1.67)
2.1–4	5.16 (3.98–6.70)		1.85 (1.69–2.03)	1.46 (1.11–1.92)
4+	12.10 (9.36–15.64)		2.06 (1.88–2.25)	2.10 (1.60–2.77)
Radiotherapy^g^				
No	REF		REF	
Yes	0.49 (0.45–0.54)		1.02 (0.98–1.07)	1.24 (1.08–1.43)
Chemotherapy^h^				
No	REF		REF	REF
Yes	0.49 (0.46–0.52)		0.14 (0.13–0.15)	0.16 (0.15–0.17)
Treatment delay				
No	REF		REF	REF
Yes	0.93 (0.90–0.96)		0.85 (0.82–0.88)	0.85 (0.82–0.88)
Tumor site for patients who underwent radiotherapy				
Radiotherapy and cecum				REF
Radiotherapy and ascending colon				1.19 (0.94–1.51)
Radiotherapy and transverse colon				1.16 (0.85–1.58)
Radiotherapy and descending colon				1.46 (1.06–2.01)
Radiotherapy and sigmoid colon				0.90 (0.75–1.07)
Radiotherapy and rectosigmoid colon				0.86 (0.72–1.02)
Radiotherapy and rectum				0.79 (0.68–0.92)
Radiotherapy and other				1.10 (0.85–1.43)
Tumor site for patients who underwent chemotherapy				
Chemotherapy and cecum				REF
Chemotherapy and ascending colon				0.99 (0.92–1.07)
Chemotherapy and transverse colon				1.00 (0.91–1.11)
Chemotherapy and descending colon				0.73 (0.65–0.83)
Chemotherapy and sigmoid colon				0.78 (0.72–0.84)
Chemotherapy and rectosigmoid colon				0.69 (0.62–0.76)
Chemotherapy and rectum				0.70 (0.64–0.76)
Chemotherapy and other				1.02 (0.94–1.12)

Abbreviation: Tvc = time‐varying covariate.

^a^ Model 1 = associations of surgery type, tumor site, and race and ethnicity with mortality outcomes adjusted for sociodemographic factors.

^b^ Model 2 = Extended Model 1 by adjusting for tumor and treatment characteristics.

^c^ Model 3 = Extended Model 2 by adjusting for the interaction of radiation therapy and chemotherapy with tumor site.

^d^ Ref = reference group.

^e^ NH = non‐Hispanic.

^f^ The values provided in Model 3 are when the patients did not undergo chemotherapy or radiotherapy.

^g^ The values provided in Model 3 represent radiotherapy for cecum cases only.

^h^ The values provided in Model 3 represent chemotherapy for cecum cases only.

### 3.3 Mediation Analysis

Mediators accounted for 51.38% (indirect effect HR: 0.92, 95% CI 0.91–0.93) of excess all‐cause mortality in Black versus White patients overall (Table 5). Variations in SES and clinicopathologic factors significantly explained 46.63% and 10.87% of excess all‐cause mortality, respectively. The Black patient versus Hispanic patient comparison identified SES as the most influential mediator, explaining 19.68% (indirect effect HR: 0.96, 95% CI 0.95–98) of excess all‐cause mortality among Black patients. The Black patient versus White patient comparison identified variations in clinicopathologic characteristics as mediating factors, while the Black patient versus Hispanic patient comparison did not. Although the magnitudes differed, the proportions mediated for CRC‐specific mortality displayed results similar to all‐cause mortality. SES was the most critical mediator in all groups, with the greatest proportion mediated (55.23%) in the Black patient versus NH Other patient comparisons. In Black patients versus White patients, variations in clinicopathologic characteristics explained 9.06% (indirect effect HR: 0.98, 95% CI: 0.96–0.99) of the excess CRC‐specific mortality. Lastly, the mediation analysis showed no evidence of mediation from treatment variations.

**TABLE 5:** Estimated proportion of racial and ethnic differences in all‐cause mortality mediated by SES and treatment factors among US adults between 2010 and 2017.

	Total effect, HR (95% CI)	Direct effect, HR (95% CI)	Indirect effect, HR (95% CI)	Proportion mediated^a^, ^b^
All‐cause mortality				
*Black (ref) vs. White patients*				
SES^c^	0.83 (0.81–0.89)	0.91 (0.88–0.94)	0.92 (0.91–0.93)	46.63%*
Clinical^d^	0.83 (0.81–0.89)	0.85 (0.82–0.88)	0.98 (0.97–0.99)	10.87%*
Treatment^e^	0.83 (0.81–0.89)	0.84 (0.81–0.86)	1.00 (0.99–1.00)	1.41%
All mediators	0.83 (0.81–0.89)	0.92 (0.89–0.94)	0.91 (0.89–0.93)	51.38%*
*Black (ref) vs. Hispanic patients*				
SES	0.83 (0.81–0.86)	0.86 (0.83–0.89)	0.96 (0.95–0.98)	19.68%*
Clinical	0.83 (0.81–0.86)	0.83 (0.81–0.86)	1.00 (0.98–1.01)	1.60%
Treatment	0.83 (0.81–0.86)	0.84 (0.81–0.86)	1.00 (0.99–1.00)	2.34%
All mediators	0.83 (0.81–0.86)	0.86 (0.83–0.88)	0.97 (0.95–0.99)	16.13%*
*Black (Ref) vs. NH other patients*				
SES	0.74 (0.71–0.77)	0.86 (0.81–0.90)	0.87 (0.83–0.90)	48.35%*
Clinical	0.74 (0.71–0.77)	0.74 (0.71–0.76)	1.01 (0.98–1.03)	−2.41%
Treatment	0.74 (0.71–0.77)	0.74 (0.71–0.77)	1.01 (0.99–1.02)	−2.80%
All mediators	0.74 (0.71–0.77)	0.82 (0.77–0.86)	0.91 (0.86–0.96)	32.10%*
CRC‐specific mortality				
*Black (Ref) vs. White patients*				
SES	0.81 (0.78–0.84)	0.86 (0.83–0.90)	0.94 (0.93–0.95)	29.92%*
Clinical	0.81 (0.78–0.84)	0.83 (0.80–0.85)	0.98 (0.96–0.99)	9.06%*
Treatment	0.81 (0.78–0.84)	0.80 (0.78–0.83)	1.01 (0.99–1.02)	−3.96%
All mediators	0.81 (0.78–0.84)	0.87 (0.84–0.90)	0.93 (0.91–0.95)	33.65%*
*Black (Ref) vs. Hispanic patients*				
SES	0.89 (0.85–0.93)	0.92 (0.88–0.96)	0.97 (0.96–0.99)	26.22%*
Clinical	0.89 (0.85–0.93)	0.89 (0.87–0.93)	1.00 (0.98–1.02)	2.00%
Treatment	0.89 (0.85–0.93)	0.90 (0.87–0.94)	0.99 (0.98–1.01)	5.44%
All mediators	0.89 (0.85–0.93)	0.91 (0.88–0.95)	0.98 (0.95–1.00)	19.33%
*Black(Ref) vs. NH Other patients*				
SES	0.80 (0.76–0.85)	0.90 (0.85–0.96)	0.88 (0.85–0.92)	55.23%*
Clinical	0.80 (0.76–0.85)	0.79 (0.75–0.83)	1.01 (0.98–1.04)	−5.75%
Treatment	0.80 (0.76–0.85)	0.79 (0.75–0.83)	1.01 (0.99–1.03)	−5.62%
All mediators	0.80 (0.76–0.85)	0.85 (0.80–0.90)	0.94 (0.89–0.99)	27.84%*

*Note:* Black used as the reference for all comparisons. The total effect represents the overall impact of race and ethnicity on mortality, encompassing all possible pathways. The direct effect isolates the impact of race and ethnicity on mortality after accounting for and excluding any influence from mediating pathways. Conversely, the indirect effect specifically captures the impact of race and ethnicity on mortality mediated through these pathways.

^a^ Proportion mediated = [(β_total − β_direct)/β_total] × 100.

^b^ We interpreted a statistically significant indirect effect as a corroboration of mediation.

^c^ SES quintiles and rurality quartiles.

^d^ Tumor grade, site, size, and grade.

^e^ Surgery type, chemotherapy, radiotherapy, and delayed treatment.

* *p* < 0.05.

## 4 Discussion

Findings from this study showed that racial and ethnic disparities in mortality among US adults who received a primary diagnosis of CRC between 2010 and 2017 were primarily influenced by SES. SES was the strongest associated mediator leading to excess all‐cause mortality among Black individuals [4, 37, 38]. Black patients had a higher risk of CRC‐specific mortality than White patients, aligning with prior studies [4, 5, 39]. Black CRC patients had a higher risk of all‐cause mortality compared to White patients, aligning with existing evidence [4, 39, 40, 41]. Patients who underwent a surgical procedure were less likely to experience all‐cause and CRC‐specific mortality. This corroborates findings that the five‐year survival rate of CRC significantly improves due to surgery [42, 43]. In our study, treatment‐related factors did not mediate all‐cause or CRC‐specific mortality. However, we observed variation in clinicopathologic factors as mediators for Black and White patients. These variations explained some of the excess CRC‐specific mortality in Black individuals, aligning with prior study findings [44]. While acknowledging the significance of clinicopathologic characteristics, it is noteworthy that their influence, though substantial, followed the overarching influence of SES. This underscores the importance of addressing and understanding patient‐level social and economic influences in any comprehensive analysis of CRC‐related outcomes, especially regarding access to prevention and treatment. Our findings emphasize the imperative of interventions targeting SES—and growing attention to health‐related social needs—to attain meaningful improvements in overall CRC‐related outcomes [45].

Most patients underwent surgical procedures aligning with CRC guideline‐concordant care recommendations [46, 47]. Timing of treatment was also an important risk factor in CRC mortality. For instance, patients who waited more than 1 month between diagnosis and the first course of treatment had a lower risk of CRC‐specific mortality. In a prior study, CRC patients who experienced 1–2 weeks of treatment delay had an increased likelihood of all‐cause mortality but not CRC‐specific mortality [48]. This may suggest that the ability to wait more than one month for CRC‐related surgery indicates lower disease severity, thus resulting in lower CRC‐specific mortality.

The most common tumor sites were the sigmoid, colon, and rectum, with more men reporting tumors in the rectum than women, in alignment with existing evidence [49, 50]. This might be attributed to the increased risk factors of CRC among men, including higher body mass index (BMI) and visceral fat deposit [51, 52]. In our study, patients with primary tumors located in the proximal colon (cecum, transverse colon) were at higher risk for all‐cause mortality and CRC‐specific mortality than patients with tumors in the rectum. Research on right‐sided colon cancer versus left‐sided colon cancer prognosis and mortality is poorly understood [53, 54]. However, evidence shows that patients with right‐sided colon cancers are often at more advanced stages, with larger and more poorly differentiated tumors [55]. Results from another study indicate that mortality rates are lower for patients with rectal cancers than for those with proximal colon cancer [56].

All‐cause mortality was greater among participants in the lowest SES quintile compared with the highest SES quintiles [57]. This is potentially attributed to the inequitable distribution of resources and access to quality healthcare according to socioeconomic class in the United States. These disparities may also be attributed to a multi‐dimensional array of issues, including behavioral risk factors, greater health‐related social needs (i.e., food insecurity, housing instability, transportation issues, interpersonal safety, and utility concerns), racism and discrimination, and generational mistrust [4, 57]. The greater all‐cause mortality risk among men compared with women may partially be explained by sex differences in the initiation and progression of CRC [51, 58, 59].

SES mediators accounted for almost half of the all‐cause mortality in Black patients compared to White patients. SES was the most significant mediator leading to excess all‐cause mortality among Black patients versus their Hispanic counterparts. There is evidence showing an association between low SES and worse outcomes among CRC patients; and in this study, Black individuals were more likely to report low SES [7, 60, 61]. Lower SES may predispose CRC patients to structural and inequitable access‐related impediments to care, worsening CRC‐specific outcomes [57].

Clinicopathologic characteristics had a lower mediating impact on all‐cause mortality than SES, the most critical mediator in all groups. However, our study appropriately corroborates other available evidence on the effects of clinicopathologic factors. For instance, tumor differentiation and stage harmed patient survival, a difference more prominent in older populations [62]. As in prior studies, ours demonstrated that tumor location significantly impacted mortality, with proximal and distal cancers showing higher mortality than rectum and rectosigmoid junction cancers [63]. Furthermore, a negative association existed between tumor size and CRC survival rate [10, 12]. CRC survival rates decreased significantly with increasing stages [64, 65]. Treatment type and a combination of treatments impacted survival rates for CRC patients [66, 67].

To our knowledge, this is the first study that quantified the extent to which SES, clinicopathologic, and treatment variations explained racial and ethnic differences in both all‐cause mortality and CRC‐specific mortality among patients diagnosed with CRC. Limitations of this study include the absence of information on the comorbidities that may have mediated all‐cause mortality and CRC‐specific mortality among patients. Another limitation of this study is the exclusion of patients with missing or unknown values for SES, clinicopathologic, or treatment‐related factors. This exclusion may have introduced bias, especially if the missing data were not randomly distributed across the population or racial and ethnic groups. This can affect the study's findings by skewing comparisons between groups, potentially underestimating disparities, and impacting the study's generalizability Despite growing evidence of an increased burden of CRC mortality among NH American Indian and Alaska Native adults, the decision to collapse these into the NH Other racial and ethnic categories, based on available data for analysis, further limited the implications of our study. Furthermore, this analysis could not consider the effect of socio‐structural factors (e.g., transportation, distance to care services, and non‐marital social support networks) that may have impacted access and utilization of therapeutic services for better CRC outcomes.

The study's findings underscore the critical need for targeted interventions to address the disproportionately higher rates of radical resection and mortality among Black patients with colorectal cancer. These disparities are driven by late‐stage presentation, socioeconomic barriers, limited access to timely and comprehensive care, and systemic inequities in healthcare delivery [4, 16, 52]. Clinically, enhancing early detection through targeted screening, ensuring equitable access to guideline‐concordant treatments, and addressing implicit biases in care provision are essential to improving outcomes [16, 33]. Furthermore, building trust through culturally sensitive communication and addressing social determinants of health, such as financial and transportation barriers, is critical to mitigating these disparities [52]. These findings highlight the urgent need for multifaceted approaches combining early intervention, equitable treatment, and systemic changes to advance health equity in CRC care [4].

## 5 Conclusion

Our study assessing racial and ethnic disparities in all‐cause and CRC‐specific mortality rates among adults in the United States reveals that SES, coupled with clinicopathologic characteristics, emerged as primary factors influencing disparately higher mortality rates among Black patients compared to White patients. This study underscores the intricate interaction between social, economic, and health‐related factors, emphasizing the need for targeted interventions addressing both patient‐level SES attributes and healthcare access to mitigate the observed disparities in mortality outcomes between racial and ethnic groups. Understanding and addressing these multifaceted determinants is crucial for advancing health equity initiatives and promoting a more just and effective healthcare system in the United States.

## Author Contributions

**Pierre Fwelo:** conceptualization, investigation, writing – original draft, methodology, writing – review and editing, software, formal analysis. **Toluwani E. Adekunle:** writing – original draft, conceptualization. **Tiwaladeoluwa B. Adekunle:** writing – original draft. **Ella R. Garza:** writing – original draft. **Emily Huang:** writing – original draft, writing – review and editing. **Wayne R. Lawrence:** writing – review and editing, writing – original draft. **Aldenise P. Ewing:** writing – original draft, writing – review and editing, conceptualization, supervision.

## Conflicts of Interest

The authors declare no conflicts of interest.

## Supporting information


**Table S1.** Surgery type grouping.
**Table S2.** Required sample size to detect different effect sizes (*w*) in a Pearson’s chi‐squared test.

## Data Availability

The data generated and/or analyzed during the current study are not publicly available due to SEER's Data use agreement and terms. Data access requests should be directly made to SEER https://seerdataaccess.cancer.gov/seer‐data‐access.
