# Supplementary material for: Differential Colorectal Cancer Mortality Across Racial and Ethnic Groups: Impact of Socioeconomic Status, Clinicopathology, and Treatment‐Related Factors
Source: Cancer Med. 2025 Mar 4;14(5):e70612. doi: 10.1002/cam4.70612 (PMC11880620; doi:10.1002/cam4.70612)
Supplement: Supplementary file 1 — Table S1. Surgery type grouping. Table S2. Required sample size to detect different effect sizes (w) in a Pearson’s chi‐squared test. [file CAM4-14-e70612-s001.docx]

**Supplementary Table S1**: Surgery type grouping

| **Region** | **Local Excision** | **Segmental Resection** | **Radical Resection** |
| --- | --- | --- | --- |
| **Colon** | Polypectomy, NOS, Excisional biopsy, Polypectomy-endoscopic, Polypectomy-surgical excision, and Electrocautery | Appendectomy (for an appendix primary only), enterocolectomy, ileocolectomy, partial colectomy, NOS, partial resection of transverse colon and flexures, and segmental resection (such as cecectomy or sigmoidectomy) | Subtotal colectomy/hemicolectomy; Total colectomy; Total proctocolectomy; Colectomy or coloproctotectomy with resection of contiguous organ(s), NOS; Colectomy, NOS |
| **Rectosigmoid** | Polypectomy, Excisional biopsy, and Electrocautery | Segmental resection; partial proctosigmoidectomy, NOS; Anterior resection; Hartmann’s operation; Low anterior resection (LAR); Partial colectomy, NOS; Rectosigmoidectomy, NOS; Sigmoidectomy | Total proctectomy; Abdominoperineal resection (A & P resection); anterior/posterior resection (A/P resection)/Miles’ operation; Rankin’s operation; Total colectomy; Total colectomy WITH ileostomy, NOS; Ileorectal reconstruction; Total colectomy WITH other pouch; Total proctocolectomy, NOS; Colectomy or proctocolectomy resection in continuity with other organs; pelvic exenteration |
| **Rectum** | Polypectomy; Excisional biopsy; Electrocautery; curette and fulguration | Segmental resection; partial proctosigmoidectomy, NOS; Anterior resection; Hartmann’s operation; Low anterior resection (LAR); Transsacral rectosigmoidectomy | Total proctectomy; Abdominoperineal resection; Total proctocolectomy, NOS; Proctectomy or proctocolectomy with resection in continuity with other organs; pelvic exenteration; Proctectomy, NOS |

**Supplementary Table S2 : Required sample size to detect different effect sizes (w) in a Pearson’s Chi-square test.**

| **Outcome** | **Main Exposure** | **DF** | **Power** | **SAMPLE SIZE: N** | | | | | |
| --- | --- | --- | --- | --- | --- | --- | --- | --- | --- |
|  |  |  |  | Effect size (w=0.05) | Effect size (w=0.1) | Effect size (w=0.2) | Effect size (w=0.3) | Effect size (w=0.4) | Effect size (w=0.5) |
| Cancer specific mortality (Yes vs no) | Race/Ethnicity | 3 | 90% | 5,669 | 1417 | 354 | 157 | 89 | 57 |
| Overall mortality (Yes vs no) | Race/ethnicity | 3 | 90% | 5,669 | 1417 | 354 | 157 | 89 | 57 |
| We used R software to determine the minimum sample size needed for 90% power to reject the null hypothesis using a chi-square test, following Cohen (1988) methodology. Cohen’s w was used as the effect size measure. The effect size is considered small if w = 0.10, medium if w = 0.30, and large if w = 0.50. Since the effect size for our study is unknown, we calculated the sample size for w values ranging from 0.05 to 0.50. Consequently, the smallest sample size required to address the research question with the smallest effect size and largest degree of freedom is 5,669 colorectal cancer patients.”  Reference: Cohen, J. (1988). *Statistical power analysis for the behavioral sciences* (2nd ed.). L. Erlbaum Associates. https://doi.org/10.4324/9780203771587 | | | | | | | | | |
